# Elevated concentrations of macrophage migration inhibitory factor in serum and cerebral microdialysate are associated with delayed cerebral ischemia after aneurysmal subarachnoid hemorrhage

**DOI:** 10.3389/fneur.2022.1066724

**Published:** 2023-01-13

**Authors:** Felix Neumaier, Christian Stoppe, Anzhela Stoykova, Miriam Weiss, Michael Veldeman, Anke Höllig, Hussam Aldin Hamou, Yasin Temel, Catharina Conzen, Tobias Philip Schmidt, Rabia Dogan, Martin Wiesmann, Hans Clusmann, Gerrit Alexander Schubert, Roel Hubert Louis Haeren, Walid Albanna

**Affiliations:** ^1^Department of Neurosurgery, RWTH Aachen University Hospital, Aachen, Germany; ^2^Institute of Radiochemistry and Experimental Molecular Imaging, Faculty of Medicine and University Hospital Cologne, University of Cologne, Cologne, Germany; ^3^Institute of Neuroscience and Medicine, Nuclear Chemistry (INM-5), Forschungszentrum Jülich GmbH, Jülich, Germany; ^4^Departments of Cardiac Anesthesiology and Intensive Care Medicine Charité, Berlin, Germany; ^5^Department of Intensive Care and Intermediate Care, RWTH Aachen University, Aachen, Germany; ^6^Department of Anesthesiology and Intensive Care Medicine, Würzburg University, Würzburg, Germany; ^7^Department of Neurosurgery, Kantonsspital Aarau, Aarau, Switzerland; ^8^Department of Neurosurgery, Maastricht University Medical Center, Maastricht, Netherlands; ^9^Department of Diagnostic and Interventional Neuroradiology, RWTH Aachen University, Aachen, Germany

**Keywords:** aneurysmal subarachnoid hemorrhage, delayed cerebral ischemia, macrophage migration inhibitory factor (MIF), cerebral microdialysis, brain

## Abstract

**Objective:**

Inflammation is increasingly recognized to be involved in the pathophysiology of aneurysmal subarachnoid hemorrhage (aSAH) and may increase the susceptibility to delayed cerebral ischemia (DCI). Macrophage migration inhibitory factor (MIF) has been shown to be elevated in serum and cerebrospinal fluid (CSF) after aSAH. Here, we determined MIF levels in serum, CSF and cerebral microdialysate (MD) at different time-points after aSAH and evaluated their clinical implications.

**Methods:**

MIF levels were measured in serum, CSF and MD obtained from 30 aSAH patients during early (EP_d1−4_), critical (CP_d5−15_) and late (LP_d16−21_) phase after hemorrhage. For subgroup analyses, patients were stratified based on demographic and clinical data.

**Results:**

MIF levels in serum increased during CP_d5−15_ and decreased again during LP_d16−21_, while CSF levels showed little changes over time. MD levels peaked during EP_d1−4_, decreased during CP_d5−15_ and increased again during LP_d16−21_. Subgroup analyses revealed significantly higher serum levels in patients with aneurysms located in the anterior *vs*. posterior circulation during CP_d5−15_ (17.3 [15.1–21.1] *vs*. 10.0 [8.4–11.5] ng/ml, *p* = 0.009) and in patients with DCI *vs*. no DCI during CP_d5−15_ (17.9 [15.1–22.7] *vs*. 11.9 [8.9–15.9] ng/ml, *p* = 0.026) and LP_d16−21_ (17.4 [11.7–27.9] *vs*. 11.3 [9.2–12.2] ng/ml, *p* = 0.021). In addition, MIF levels in MD during CP_d5−15_ were significantly higher in patients with DCI *vs*. no DCI (3.6 [1.8–10.7] *vs*. 0.2 [0.1–0.7] ng/ml, *p* = 0.026), while CSF levels during the whole observation period were similar in all subgroups.

**Conclusion:**

Our findings in a small cohort of aSAH patients provide preliminary data on systemic, global cerebral and local cerebral MIF levels after aSAH and their clinical implications.

**Clinical trial registration:**

ClinicalTrials.gov, identifier: NCT02142166.

## 1. Introduction

Despite aggressive multidisciplinary treatment, aneurysmal subarachnoid hemorrhage (aSAH) is still associated with a high overall morbidity and mortality (1). In addition to early brain injury (EBI), a significant number of aSAH patients develop delayed cerebral ischemia (DCI); a complication that can result in secondary brain injury (2). Although long attributed to angiographic vasospasm, DCI is now recognized to reflect a cascade of events that also comprises microvascular dysfunction due to inflammation, oxidative stress and other factors (3–5). Timely treatment of DCI is essential to prevent long-term impairments but its detection remains a diagnostic challenge, especially in unconscious patients. For example, because DCI also occurs in the absence of angiographic vasospasm (6), measurements utilizing near-infrared spectroscopy (7, 8) or transcranial Doppler (9, 10) often lack sufficient specificity for its faithful detection.

Given the emerging role of inflammation in the pathophysiology of aSAH, inflammatory cytokines are increasingly recognized as potential predictive and/or diagnostic biomarkers for DCI in poor grade aSAH patients (11). Macrophage migration inhibitory factor (MIF) is a cytokine expressed by a broad range of cell types (including immune cells, endocrine cells, epithelial and endothelial cells, as well as neurons and glial cells) and secreted in response to pathophysiological stimuli such as hypoxia, surgical stress or infections. MIF is an upstream regulator of the immune response and key mediator of local and systemic inflammatory responses (12, 13) that has been implicated in numerous immune-mediated pathologies (14–16). Previous studies indicate that elevated levels of MIF in serum and cerebrospinal fluid (CSF) may be associated with DCI and poor functional outcomes after aSAH (17–19). However, no previous studies have examined the relationship between systemic (i.e., serum) and cerebral (i.e., CSF) MIF concentrations in the same group of patients, which could provide insight into the exact origin of MIF released in response to aSAH. In addition, there is still a lack of data on local MIF concentrations in the brain and their potential association with DCI and functional impairments after aSAH. Therefore, we evaluated MIF levels in serum, CSF and cerebral microdialysate (MD) in patients after aSAH and evaluated their clinical significance.

## 2. Methods

### 2.1. Ethical considerations

The study protocol was approved by the local ethics committee (EK062/14), and written consent for study inclusion was obtained from all patients or their legal representatives. This cohort observational study has been registered at ClinicalTrials.gov (NCT02142166) as part of a larger scale prospective data collection.

### 2.2. Patient population and demographics

The present study is based on 30 aSAH patients treated at our facility between 12/2014 and 06/2017 who met the following inclusion criteria: (1) age>18 years, (2) availability of an external ventricular drainage for CSF collection and/or cerebral catheters for MD collection (see below), and (3) no foreseeable early mortality due to brain stem injury. Demographic data obtained on admission or during in-hospital treatment included sex, age, body mass index (BMI), blood pressure, smoking status, aneurysm location, treatment modality, clinical condition on admission according to the Hunt & Hess (HH) grading scale, radiological severity on admission according to the modified Fisher Scale (mFS) and clinical outcome after 12 months according to the extended Glasgow Outcome Scale (GOSE). Clinical outcome was assessed by an independent physician based on clinical evaluation in the outpatient clinic or the clinical status compiled from medical reports.

### 2.3. Standard treatment procedure

After aneurysms were secured by surgical clipping or endovascular treatment, prophylactic enteral nimodipine (6 × 60 mg) was applied as tolerated (20), and the patients were monitored for the following signs of DCI: In awake patients, DCI was diagnosed based on the criteria for clinical deterioration defined by Vergouwen (2), which comprise appearance of new focal neurological deficits or a decrease in Glasgow Coma Scale by ≥2 points for ≥1 h. In unconscious patients, DCI was defined as cerebral metabolic (lactate/pyruvate ratio ≥ 40) or oxygenation (p_ti_O_2<_ 10 mmHg) crisis or appearance of new CT perfusion deficits if other causes could be ruled out. To this end, invasive neuromonitoring was performed according to recent consensus recommendations (7) by measurement of brain tissue oxygenation with Neurovent PTO catheters (Raumedic AG, Helmbrechts, Germany) and cerebral metabolism with 71 high cut-off microdialysis catheters (μdialysis, Stockholm, Sweden), which were perfused at 0.3 μl/min with standard perfusion fluid. Brain oxygenation probes and microdialysis catheters were advanced about 3–4 cm subcortically *via* a small craniostomy and after dural perforation, so that they terminated within the vascular territory of the aneurysm, and were held in place by a bolt affixed within the craniostomy (21). Samples were collected at least every 3 h and analyzed as described previously (21).

The first line of treatment for DCI involved induction of euvolemic hypertension by intravenous noradrenaline infusion to raise systolic arterial blood pressure to ≥180 mmHg. When there was no improvement, relevant hypoperfusion and vasoconstriction were verified by cerebral angiography and/or perfusion CT and endovascular rescue treatment was performed as described previously (21, 22). Cerebral infarctions during ongoing DCI or diagnosed as first sign of DCI and confirmed by at least two imaging modalities were considered DCI-related infarctions.

### 2.4. Sampling and data collection

Serum (7 ml) and CSF (1 ml, obtained from the proximal access point of a ventricular drain) were collected from every patient between days 1–21 (day 1–3: once; day 4–21: every third day). Whenever possible, MD (30–120 μl) was collected every 6 hours following a stabilization period of 24 h after catheter insertion. Vials containing MD samples were retained in designated storage racks. Blood samples were immediately centrifuged and serum and CSF were pipetted into suitable polypropylene cryotubes (VWR International, Darmstadt, Germany). All samples were permanently deep-frozen at −80°C, protected from light, and quantitatively analyzed for MIF levels at a “Good Laboratory Practice” (GLP) certified laboratory using a customized U-PLEX^®^ Biomarker Group 1 Multiplex Assay from MSD (Meso Scale Discovery^®^, Cat-No: K15067L-2).

Based on the typical time-course for occurrence of EBI and DCI, we defined three time windows in reference to the initial insult on day 0 for data danalysis, which comprised an early phase (days 1–4: EP_d1−4_) for reflection of initial injury and aSAH severity, a critical phase (days 5–15: CP_d5−15_) characterized by high susceptibility to DCI, and a late phase (days 16–21: LP_d16−21_) corresponding to the end of ICU and/or DCI treatment. If more than one measurement was available for a patient in a given period, all measurements in this period were used to calculate a mean value. In some cases, the critical phase was further subdivided into an early critical (CP_d5−8_), intermediate critical (CP_d9−12_) and late critical (CP_d13−15_) phase to examine the time-course of changes during days 5–15.

### 2.5. Subgroup analysis

For subgroup analysis, patients were stratified into groups according to sex, age (<59 years *vs*. ≥59 years based on the median), smoking status (smoker *vs*. non-smoker), BMI (BMI<26 *vs*. BMI≥26 based on the median), arterial hypertension (hypertension *vs*. no hypertension), systemic infection diagnosed based on clinical signs of sepsis (infection *vs*. no infection), clinical condition (good grade = HH_1−3_ *vs*. poor grade = HH_4−5_) and radiological classification on admission (good grade = mFS_1−2_ *vs*. poor grade = mFS_3−4_), treatment modality (endovascular treatment *vs*. surgical clipping), aneurysm location (anterior *vs*. posterior circulation), occurrence of DCI (DCI *vs*. no DCI) or DCI-related infarction (infarction *vs*. no infarction), and clinical outcome after 12 months (favorable = GOSE_5−8_ *vs*. unfavorable = GOSE_1−4_).

### 2.6. Statistical methods

After testing for normality *via* the Shapiro-Wilk test, continuous parametric data were compared using *T*-tests, while the Mann-Whitney *U*-test or Wilcoxon signed-rank test were used for non-parametric data, as applicable. Nominal data were tested between groups using the Fisher's exact test or Chi^2^ test. For categorical variables, data are given as numbers and percentages. Parametric values are expressed as mean ± standard deviation (SD), whereas non-parametric values are expressed in terms of median values [25th−75th percentile]. Boxplots show median, 25th and 75th percentile (box), minimum and maximum (whiskers) and individual data points (dots). Because MD levels of MIF often ranged from very small (<0.1 ng/ml) to very large (>37 ng/ml) values, a log scale was used in some of the corresponding figures to better visualize all of the data points shown. Correlation analysis was performed using the Spearman's rank correlation coefficient (r_s_) as a non-parametric measure of rank correlation. Statistical significance was set at p<0.05 and statistical results with *p* ≤ 0.1 were considered as trend. All analyses were performed using IBM^®^ SPSS^®^ Statistics V22.0 (IBM, Armonk, NY, USA), Microsoft Excel 2010 (Microsoft, Redmond, USA) or Minitab 17.1.0 (Minitab Inc., Pennsylvania, USA).

## 3. Results

### 3.1. Patients

A total of 30 aSAH patients with a median age of 59 [50–64] years and a female:male ratio of 22 (73%) to 8 (27%) were included in the present study. A summary of their demographic data is provided in Table 1. Seventeen (57%) of the patients developed DCI, which occurred between day 3 and 10 (median: day 7 [5-9]) and progressed to infarction in six (35%) of these patients. Clinical outcome after 12 months was favorable (GOSE_5−8_) in 16 (53.3%) and unfavorable (GOSE_1−4_) in 10 (33.3%) patients, while no follow-up data were available for the remaining four patients (13.3%). Clinical outcome was independent of sex (*p* = 1.000), age (*p* = 0.756), BMI (*p* = 0.248), arterial hypertension (*p* = 0.701), smoking status (*p* = 0.340), systemic infection (*p* = 0.248), treatment modality (*p* = 0.234), clinical (*p* = 0.339) or radiological (*p* = 0.190) status on admission according to HH or mFS, respectively, development of DCI (*p* = 0.248) or DCI-related infarctions (*p* = 0.163) and ventilation time (*p* = 0.516). Anterior circulation aneurysms were more frequent (70%) and tended to be associated with unfavorable clinical outcomes (*p* = 0.087).

**Table 1:** Patient characteristics and their relation to clinical outcome after 12 months.

**Patient characteristic | *n* (%)**	**All**	**missing data**	**Favorable** **outcome (GOSE_5−8_)**	**Unfavorable outcome (GOSE_1−4_)**	***p*-value^*a*^**
All patients	30 (100%)	4 (13.3%)	16 (53.3%)	10 (33.3%)	
**Sex**					1.000
Female	22 (73%)	2 (50%)	12 (75%)	8 (80%)	
Male	8 (27%)	2 (50%)	4 (25%)	2 (20%)	
**Age**					0.756
< 59 years	14 (47%)	2 (50%)	7 (44%)	5 (50%)	
≥59 years	16 (53%)	2 (50%)	9 (56%)	5 (50%)	
Median [q1–q3]	59 [50–64]	58 [49–63]	59 [51–62]	57 [47–71]	0.958
**Body mass index (BMI)**					0.248
BMI < 26	14 (47%)	2 (50%)	9 (56%)	3 (30%)	
BMI ≥26	16 (53%)	2 (50%)	7 (44%)	7 (70%)	
Median [q1–q3]	26 [22–28]	26 [24–27]	25 [22–26]	27 [25–31]	0.094
**Arterial hypertension**					0.701
No	14 (47%)	2 (50%)	8 (50%)	4 (40%)	
Yes	16 (53%)	2 (50%)	8 (50%)	6 (60%)	
**Smoker**					0.340
No	23 (77%)	2 (50%)	14 (88%)	7 (70%)	
Yes	7 (23%)	2 (50%)	2 (12%)	3 (30%)	
**Infection**					0.248
No	20 (67%)	4 (100%)	12 (75%)	4 (40%)	
Yes	10 (33%)	0 (0%)	4 (25%)	6 (60%)	
**Aneurysm location**					0.087
Anterior circulation	21 (70%)	4 (100%)	8 (50%)	9 (90%)	
Posterior circulation	9 (30%)	0 (0%)	8 (50%)	1 (10%)	
**Treatment modality**					0.234
Clipping	11 (37%)	2 (50%)	4 (25%)	5 (50%)	
Endovascular treatment	19 (63%)	2 (50%)	12 (75%)	5 (50%)	
**Hunt and Hess grade (HH)**					0.339
Good grade (HH_1−3_)	20 (67%)	4 (100%)	11 (69%)	5 (50%)	
Poor grade (HH_4−5_)	10 (33%)	0 (0%)	5 (31%)	5 (50%)	
**Modified Fisher Scale (mFS)**					0.190
Good grade (mFS_1−2_)	7 (23%)	0 (0%)	6 (37%)	1 (10%)	
Poor grade (mFS_3−4_)	23 (77%)	4 (100%)	10 (63%)	9 (90%)	
**Delayed cerebral ischemia (DCI)**					0.248
no DCI	13 (43%)	1 (25%)	9 (56%)	3 (30%)	
DCI	17 (57%)	3 (75%)	7 (44%)	7 (70%)	
**DCI-related infarction**					0.592
DCI only	11/17 (65%)	3/3 (100%)	5/7 (71%)	3/7 (43%)	
DCI-related infarction	6/17 (35%)	0/3 (0%)	2/7 (29%)	4/7 (57%)	
**Ventilation time [days]**					0.516
Median [q1–q3]	26 [18–37]	39 [35–40]	24 [18–27]	25 [18–36]	

a *p*-values indicate if there was a significant difference between the respective subgroups with regard to clinical outcome (e.g., favorable vs. unfavorable outcome). q1, 25th percentile; q3, 75th percentile.

### 3.2. Changes in serum, CSF and MD MIF levels after aSAH

**Figure 1:**
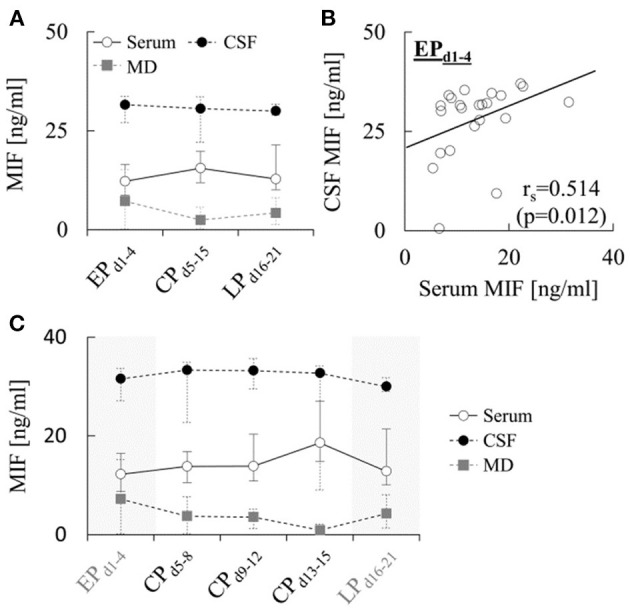
Concentration of MIF in different compartments after aSAH. **(A)** Concentration of macrophage migration inhibitory factor (MIF) measured in the indicated compartments during the early (EP_d1−4_), critical (CP_d5−15_) and late (LP_d16−21_) phase after aneurysmal subarachnoid hemorrhage (aSAH). In total, at least one sample for quantification of MIF levels during EP_d1−4_, CP_d5−15_ and LP_d16−21_ was available from 26, 19 and 26 (serum), 23, 15 and 11 (CSF) and 9, 20 and 8 (MD) patients, respectively (see Supplementary Tables 1–3). Data are shown as median [25th−75th percentile]. Statistical significance of changes over time was assessed using a repeated-measures ANOVA. **(B)** Correlation between MIF concentrations in CSF and serum during the early phase. Correlation analysis was performed using the Spearman's rank correlation coefficient. **(C)** Same data as in A, but with measurements obtained between days 5 and 15 subdivided into three sub-intervals to illustrate temporal changes during the critical phase. Note that the number of patients for which samples in these sub-intervals were available was accordingly lower (*n* = 10–12, 7–10, and 10–16 per sub-interval for serum, CSF and MD, respectively) than the numbers for CP_d5−15_ given in **A** and Supplementary Tables 1–3.

A comparison of MIF levels determined in serum, CSF and MD during the different phases after hemorrhage is provided in Figure 1A. Median serum levels increased from 12.2 [8.7–16.4] ng/ml during EP_d1−4_ to 15.5 [11.9–19.8] ng/ml during CP_d5−15_ (*p* = 0.281), and decreased again to 12.8 [10.1–21.4] ng/ml during LP_d16−21_ (*p* = 0.078). The levels in CSF were roughly two-fold higher and showed little changes over time, with median levels of 31.6 [27.1–33.7] ng/ml during EP_d1−4_, 30.6 [22.1–33.6] ng/ml during CP_d5−15_ (*p* = 0.917) and 30.0 [29.0–31.8] ng/ml during LP_d16−21_ (*p* = 0.575). Finally, MD levels of the cytokine during EP_d1−4_ (7.2 [0.2–15.1] ng/ml) were almost five times lower than CSF levels, and further decreased to 2.5 [0.3–5.7] ng/ml during CP_d5−15_ (*p* = 0.091), but increased again to 4.2 [1.3–8.1] ng/ml during LP_d16−21_ (*p* = 0.180). None of the changes over time in any of the compartments reached statistical significance. However, correlation analysis revealed a significant positive correlation between MIF levels in serum and CSF during EP_d1−4_ (Spearman r_s_=0.514, *p* = 0.012, Figure 1B), while no significant correlations were detected between early serum and MD (*r*_s_ = 0.429, *p* = 0.397) or CSF and MD levels (*r*_s_ = 0.400, *p* = 0.600). In addition, no significant correlations between MIF levels in different compartments were detected during CP_d5−15_ or LP_d16−21_. Further subdivision of CP_d5−15_ into three time-intervals (Figure 1C) showed little change in CSF MIF levels over time, while the values in serum and MD showed a progressive increase and decrease, respectively, during the first 15 days.

### 3.3. Subgroup analyses

**Figure 2:**
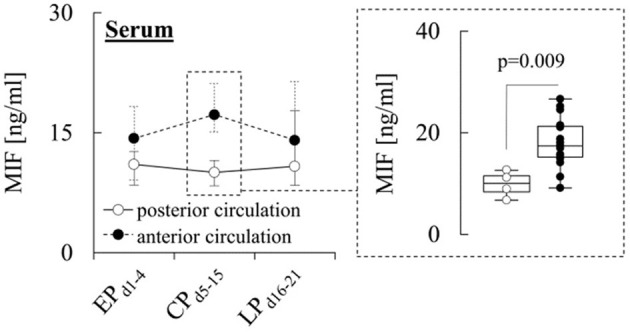
Serum MIF levels and aneurysm location. Concentration of macrophage migration inhibitory factor (MIF) in serum from patients with aneurysms located in the anterior or posterior circulation during the early (EP_d1−4_), critical (CP_d5−15_) and late (LP_d16−21_) phase after aneurysmal subarachnoid hemorrhage (aSAH). Data are shown as median [25th−75th percentile] and (for significant differences) as boxplots and individual values (see inset). Statistical significance was assessed using the Mann-Whitney *U*-test. For details on the exact number of patients per time-point see Supplementary Table 1.

When comparing the different subgroups, a prominent difference in serum MIF levels was observed for patients stratified according to aneurysm location. In particular, during CP_d5−15_, patients with anterior circulation (AC) aneurysms exhibited significantly (*p* = 0.009) higher serum levels (17.3 [15.1–21.1] ng/ml) than patients with posterior circulation (PC) aneurysms (10.0 [8.4–11.5] ng/ml) (Figure 2), while CSF and MD levels were similar in both subgroups (Supplementary Tables 2, 3).

**Figure 3:**
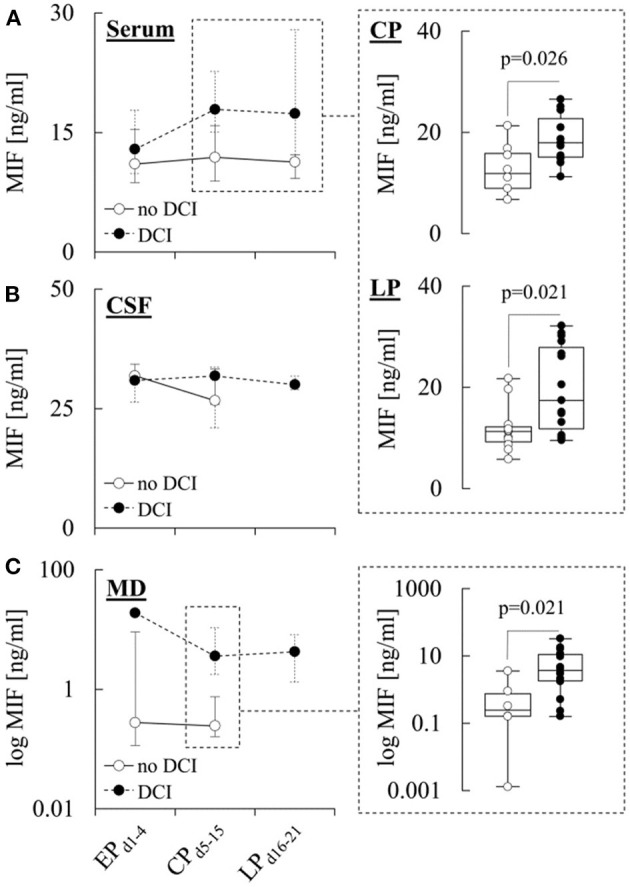
MIF levels in different compartments and the development of DCI. Concentration of macrophage migration inhibitory factor (MIF) in **(A)** serum, **(B)** cerebrospinal fluid (CSF), and **(C)** microdialysate (MD) from patients with or without delayed cerebral ischemia (DCI) during the early (EP_d1−4_), critical (CP_d5−15_) and late (LP_d16−21_) phase after aneurysmal subarachnoid hemorrhage (aSAH). Data are shown as median [25th−75th percentile] and (for significant differences) as boxplots and individual values (see insets). Statistical significance was assessed using the Mann-Whitney *U*-test. For details on the exact number of patients per time-point see Supplementary Tables 1–3.

In Figure 3, MIF levels in patients with and without DCI are illustrated. Serum levels of the cytokine were similar in both subgroups during EP_d1−4_, but significantly higher in patients with DCI during CP_d5−15_ (17.9 [15.1–22.7] ng/ml *vs*. 11.9 [8.9–15.9] ng/ml in patients without DCI, *p* = 0.026) and LP_d16−21_ (17.4 [11.7–27.9] ng/ml *vs*. 11.3 [9.2–12.2] ng/ml in patients without DCI, *p* = 0.021) (Figure 3A). In addition, MIF levels in MD samples from patients with DCI were significantly higher during CP_d5−15_ (3.6 [1.8–10.7] ng/ml *vs*. 0.2 [0.1–0.7] ng/ml in patients without DCI, *p* = 0.021) (Figure 3C), while there were no differences in CSF levels between the two subgroups (Figure 3B). There were also no differences between MIF levels in any of the compartments when patients with DCI were further stratified into patients with DCI only and patients with DCI-related infarction (Supplementary Tables 1–3).

A tendency for higher serum levels during CP_d5−15_ was observed in patients with unfavorable clinical outcomes, although these differences did not reach statistical significance (Supplementary Figure 1A). Interestingly however, after stratification according to outcome, the correlation between serum and CSF MIF levels during EP_d1−4_ (i.e., Figure 1B) was no longer observed in patients with favorable outcomes but became even stronger in patients with unfavorable outcomes (Supplementary Figure 1A inset). In addition, there were again no differences between CSF MIF levels (Supplementary Figure 1B), but MD levels of the cytokine during CP_d5−15_ tended to be higher in patients with unfavorable outcomes (Supplementary Figure 1C) and to be negatively correlated with the GOSE score (Supplementary Figure 1C inset).

When the measurements during CP_d5−15_ were further subdivided into three time-periods, the progressive increase in serum MIF shown in Figure 1C appeared to be almost exclusively attributable to patients with AC aneurysms, while serum levels in patients with PC aneurysms actually decreased over time and were significantly lower on days 9–12 (9.0 [6.8–11.5] ng/ml *vs*. 18.9 [14.1–21.6] ng/ml in patients with AC aneurysms, *p* = 0.042, Supplementary Figure 2A). In addition, patients with DCI exhibited consistently higher MD MIF levels during the first 12 days after hemorrhage, although a significant difference was only observed at the start of the critical phase (days 5–8), when MD MIF levels amounted to 5.7 [3.9–16.1] ng/ml in patients with DCI compared to 0.2 [0.1–0.3] ng/ml in patients without DCI (*p* = 0.007, Supplementary Figure 2D).

Finally, MIF levels in serum, CSF or MD were neither associated with sex, hypertension, smoking status or treatment modality, nor with the clinical or radiological status on admission, although there was a tendency for higher serum levels in patients with poor radiological status throughout the observation period (Supplementary Tables 1–3). Likewise, even though clinical signs of sepsis were associated with a tendency for higher serum levels during CP_d5−15_ and LP_d16−21_ (Supplementary Table 1), the levels in CSF and MD were similar in patients with or without infection (Supplementary Tables 2, 3).

## 4. Discussion

In the present study, we examined temporal changes of MIF levels in serum, CSF, and MD during the first 3 weeks after hemorrhage and their relation to disease progression and outcome. Our findings indicate that anterior circulation (AC) aneurysms may be associated with higher MIF levels in serum and that higher serum and especially MD MIF levels during the critical phase may be associated with DCI. In addition, they provide preliminary data on differences between systemic (i.e., serum), global cerebral (i.e., CSF) and local cerebral (i.e., MD) MIF levels, with potential implications for its origin and pathophysiological relevance.

### 4.1. Changes in systemic MIF levels after aSAH

Unlike most other cytokines, MIF is constitutively expressed in various tissues, most notably the pituitary and adrenal glands, from which it can be released into circulation in response to inflammatory, hypoxic and other stress stimuli (13, 23, 24). Previous studies have demonstrated that ischemic stroke (25, 26), intracerebral hemorrhage (27) and aSAH (17, 18) are associated with acute increases of circulatory MIF levels, which is in line with our observation that serum MIF levels during EP_d1−4_ (12.2 [8.7–16.4] ng/ml) exceeded the upper limit of 10 ng/ml typically observed in healthy subjects (17, 28). Interestingly, previous studies reported median serum MIF levels of 30.1 [19.1–37.9] ng/ml (17) and 22.3 [17.4–27.0] ng/ml (18) in aSAH patients immediately at hospital admission, which is roughly two-fold higher than the values we observed during EP_d1−4_. Considering that serum MIF levels after ischemic stroke have been shown to peak within the first 24 h after ictus (25, 26, 29), this difference could be attributable to our sampling window for EP_d1−4_, which comprised the first 4 days after hemorrhage but not the day of aSAH onset.

Furthermore, we found a significant positive correlation between serum and CSF MIF levels during EP_d1−4_ (Figure 1B), possibly indicating that intrathecal MIF release could be involved in the acute elevation of circulating MIF after aSAH. Thus, assuming a physiological serum concentration of 6–10 ng/ml (17, 28), a pathological CSF concentration of 30 ng/ml, and a 1:10 dilution after CSF flow into venous blood (30), the early serum levels of 12.2 [8.7–16.4] ng/ml we observed could well be accounted for by CSF-related changes of MIF concentrations in blood, especially given that early impairments of blood-brain- and blood-CSF-barrier are well-known features of aSAH (31, 32), which could be further aggravated by MIF itself (33). The fact that after stratification based on outcome, a significant correlation was only observed in patients with poor clinical outcome (as shown in the Supplementary material) is in line with this assumption, since early blood-brain-barrier dysfunction has been shown to predict neurological outcomes after aSAH (34).

In addition, we found that aSAH is associated with a further delayed increase of serum MIF levels during CP_d5−15_ (Figure 1C). During this phase, there was no longer a correlation between CSF and serum levels. We also noted that differences in serum MIF levels between patients with and without signs of clinical sepsis were limited and non-significant, as shown in the Supplementary material. While this might suggest that systemic infection is not the main stimulus for increased circulating MIF concentrations in aSAH patients, it could also simply reflect the small sample size of our study.

Interestingly, the increase during CP_d5−15_ appeared to be restricted to patients with AC aneurysms, whereas serum MIF levels in patients with PC aneurysms actually decreased during the first 2 weeks after ictus, as shown in the Supplementary material. Blood supply to the pituitary gland depends on vessels from the AC (35) and the MIF gene has been shown to contain several hypoxia-responsive elements in its promoter (29, 36, 37). This makes it tempting to speculate that hypoxia-induced up-regulation of MIF expression in the pituitary gland could be involved in the increase of serum MIF levels during CP_d5−15_. However, given that the AC is responsible for more than 80% of the brain's blood supply (38), the increase of serum levels in these patients could also reflect other factors, such as a more extensive hypoperfusion of brain tissue during the initial insult. Moreover, based on the small number of patients, of which the majority (70%) had AC aneurysms, further investigations are needed to firmly establish if there are associations between circulating MIF and aneurysm location.

### 4.2. Changes in local and global cerebral MIF levels after aSAH

In contrast to the time-course of changes in serum, MD MIF levels peaked during EP_d1−4_ and progressively decreased during the first 2 weeks after hemorrhage (Figure 1C), which is reminiscent of the stereotyped response of various brain cytokines to injury observed after aSAH and traumatic brain injury (39). In addition, MD MIF levels were independent of the levels in CSF or serum, suggesting that they could reflect local cytokine release from glial, neuronal and/or immune cells, which have all been demonstrated to constitutively and/or inducibly express MIF (13, 40, 41). Clearance of the cytokine from brain interstitial fluid into CSF could possibly account for the decrease of MD MIF levels during the first 2 weeks. However, CSF levels were at least four to five times higher than the values in MD (Figure 1A), suggesting that cleared MIF either accumulates in the CSF or that another source of MIF release into the CSF exists. For example, previous animal studies found that cells of the choroid plexus exhibit the strongest baseline immunoreactivity for MIF in the rat brain and that stress stimuli result in its immediate release into the CSF (40). Resident or infiltrating monocytes/macrophages could form another source for release of MIF, thereby contributing to the sustained elevation of CSF MIF levels after aSAH.

It should be noted that, in contrast to our findings, a previous smaller observational study observed peak CSF MIF levels on day 9 after aSAH and found them to be significantly higher in patients with systemic infection and/or DCI (19). We can only speculate that this might be related to differences in the study populations, treatment protocols, sample handling and analysis procedures or other factors.

### 4.3. Association of MIF levels with DCI

Serum MIF levels during CP_d5−15_ and LP_d16−21_ were also significantly higher in patients with DCI (Figure 3A). Interestingly, and in contrast to the changes in MD (see below), serum MIF levels in patients with and without DCI started at similar levels but grew differently during the study period and peaked at the end of CP_d5−15_, as shown in the Supplementary material. This may suggest that DCI preceded the observed increase in serum levels, and that the higher levels in DCI patients simply reflect their more severe status and/or the requirement for more intense treatments (like induced hypertension or invasive ventilation). In line with this assumption, we found no evidence that the delayed increase of serum MIF levels was due to local or global cerebral release of the cytokine, suggesting that it mainly reflected peripheral release of MIF, which could well be aggravated by invasive treatments and infective or other complications. More importantly, our findings also indicate that local (MD) rather than global (CSF) cerebral levels of the cytokine could be associated with the development of DCI, as reflected in significantly higher MD MIF levels in patients with DCI during CP_d5−15_ (Figure 3C). When the samples obtained during CP_d5−15_ were subdivided into three time-periods, significantly higher MD MIF levels in patients with DCI were observed on days 5–8, as shown in the Supplementary material. This coincided almost exactly with the occurrence of DCI in our study (median: day 7 [5-9]).

To the best of our knowledge, data on the pathophysiological role of MIF in the brain is still sparse. For example, previous findings on the effects of MIF on neuronal function in different stroke models are conflicting, with some studies reporting that the cytokine is up-regulated and aggravates neurologic deficits by promoting cell death (42, 43) and/or increasing blood-brain-barrier permeability (33), while others found that it is down-regulated and may have neuroprotective functions (44, 45). With regards to aSAH, our findings argue for a local increase of MIF levels in the brain of patients with DCI, but they provide no clear-cut evidence regarding the pathophysiological relevance. Thus, higher MD MIF levels in these patients could be interpreted in terms of a detrimental role of the cytokine which increases the susceptibility to DCI, or in terms of a compensatory up-regulation that serves neuroprotective functions and is more pronounced in affected patients.

### 4.4. Limitations

Given the limits of a small exploratory study, several limitations have to be carefully considered for the interpretation of our findings. The sample size in our study was notably small and not based on any formal power calculations, and we did not correct for multiple statistical comparisons. The availability of CSF and/or MD samples was a requirement for inclusion in this study, which may have resulted in an over-representation of more severely affected patients, as reflected in the relatively high incidence of DCI (Table 1). Even more importantly, samples from the different compartments were not always available for all patients, which may have affected the observed time-course of changes, especially with regard to EP_d1−4_ and LP_d16−21_. Thus, while during CP_d5−15_, at least one MD sample was available from the majority (20/30 = 67%) of patients, the number of patients with MD samples during EP_d1−4_ and LP_d16−21_ were only 9 (30%) and 8 (27%), respectively. In addition, analysis of MD samples may have been influenced by factors such as variable catheter placement or MIF recovery. As such, our data should be regarded as preliminary and interpreted with due caution until they can be validated by human studies with adequate power. Moreover, our findings provide no information on causal relationships between MIF levels and aneurysm location, DCI or clinical outcome, which needs to be addressed in appropriate animal models.

## 5. Conclusion

Our findings in a small cohort of aSAH patients provide first data on differences between systemic, global cerebral and local cerebral MIF levels after aSAH, and their potential relation to aneurysm location and DCI. Larger clinical trials and studies with animal models will be required to validate our findings and explore potential causal relationships between MIF levels and disease progression.

## Data availability statement

The original contributions presented in the study are included in the article/Supplementary material, further inquiries can be directed to the corresponding author.

## Ethics statement

The studies involving human participants were reviewed and approved by the local ethics committee of the RWTH Aachen (EK062/14). The patients/participants provided their written informed consent to participate in this study.

## Author contributions

Conceived, designed, and performed the experiments: FN, CS, AS, and WA. Data acquisition: AS, MWe, MV, RD, CC, and WA. Analysis and interpretation of data: FN, CS, AS, YT, RH, and WA. First drafting of the manuscript and illustrations: FN and WA. Critical review of the manuscript: CS, HH, YT, MWi, HC, GS, RH, AH, TS, CC, FN, and WA. The final manuscript was approved by all authors.
