# Supplementary material for: Elevated concentrations of macrophage migration inhibitory factor in serum and cerebral microdialysate are associated with delayed cerebral ischemia after aneurysmal subarachnoid hemorrhage
Source: Front Neurol. 2023 Jan 13;13:1066724. doi: 10.3389/fneur.2022.1066724 (PMC9880331; doi:10.3389/fneur.2022.1066724)
Supplement: Supplementary file 1 [file Data_Sheet_1.docx]

**Supplemental Material**

**Suppl. Figure 1.** Dependence of MIF levels in different compartments on long-term clinical outcome.

Concentration of macrophage migration inhibitory factor (MIF) in (**A**) serum, (**B**) cerebrospinal fluid (CSF), and (**C**) microdialysate (MD) from patients with a favorable (GOSE_5-8_) or unfavorable (GOSE_1-4_) clinical outcome after 12 months during the early (EP_d1-4_), critical (CP_d5-15_) and late (LP_d16-21_) phase after aneurysmal subarachnoid hemorrhage (aSAH). Data are shown as median [1.quartile-3.quartile]. Inset in A compares the correlation between serum and CSF MIF levels during the early phase in patients with a favorable (top) or unfavorable (bottom) clinical outcome, while inset in C shows the correlation between MIF levels in MD during the critical phase and extended Glasgow Outcome Scale (GOSE). Correlation analysis was performed using the Spearman’s rank correlation coefficient.





**Suppl. Figure 2.** Time course of changes in serum and MD MIF levels during the critical phase.

(**A**-**C**) Comparison of changes in serum macrophage migration inhibitory factor (MIF) levels between patients stratified according to (**A**) aneurysm location, (**B**) development of delayed cerebral ischemia (DCI) or (**C**) clinical outcome after 12 months according to the extended Glasgow Outcome Scale (GOSE). (**D**&**E**) Comparison of changes in microdialysate (MD) MIF levels between patients stratified according to (**D**) development of DCI or (**E**) clinical outcome after 12 months. Data are shown as median [25^th^ – 75^th^ percentile] and (for significant differences) as boxplots and individual values (see insets). Statistical significance was assessed using the Mann-Whitney U test. Note that significant differences are only indicated for the three sub-intervals during the critical phase (CP_d5-8_, CP_d9-12_, CP_d13-15_). For significant differences during the early (EP_d1-4_) and late (LP_d16-21_) phase and details on the exact number of patients per time-point, see **Suppl. Tab. 1-3**.

**Suppl. Table 1.** Subgroup comparison of MIF levels in serum after aSAH.

| **Serum MIF [ng/ml]** | **Early phase (EP_d1-4_)** | | | **Critical phase (CP_d5-15_)** | | | **Late phase (LP_d16-21_)** | | |
| --- | --- | --- | --- | --- | --- | --- | --- | --- | --- |
|  | **n** | **Median**  **[1.q-3.q]** | **p-value** | **n** | **Median**  **[1.q-3.q]** | **p-value** | **n** | **Median**  **[1.q-3.q]** | **p-value** |
| **All patients^a^** | 26 | 12.2 [8.7-16.4] | | 19 | 15.5 [11.9-19.8] | | 26 | 12.8 [10.1-21.4] | |
| **Sex** |  |  | 0.267 |  |  | 0.484 |  |  | 0.977 |
| Male | 8 | 16.2 [8.9-17.8] | | 4 | 13.3 [11.7-16.7] | | 7 | 12.6 [11.5-14.1] | |
| Female | 18 | 11.8 [7.8-14.3] | | 15 | 16.8 [13.0-19.8] | | 19 | 14.8 [10.0-23.9] | |
| **Age** |  |  | 0.181 |  |  | 0.554 |  |  | 0.939 |
| < 59 years | 12 | 10.6 [8.1-14.9] | | 7 | 12.6 [10.0-21.2] | | 13 | 11.3 [9.8-26.1] | |
| ≥ 59 years | 14 | 13.3 [10.8-18.4] | | 12 | 16.2 [14.6-19.2] | | 13 | 13.0 [11.4-19.6] | |
| **Body mass index (BMI)** |  | **0.040** | |  | 0.462 | |  | 0.663 | |
| BMI < 26 | 11 | 10.3 [7.7-11.9] | | 9 | 14.8 [8.9-20.9] | | 13 | 11.7 [10.1-26.6] | |
| BMI ≥ 26 | 15 | 14.9 [11.3-18.5] | | 10 | 16.4 [14.3-18.4] | | 13 | 13.0 [11.3-17.4] | |
| **Arterial hypertension** |  |  | 0.979 |  |  | 0.142 |  |  | 0.489 |
| No | 11 | 12.1 [9.9-15.5] | | 9 | 11.2 [8.9-18.6] | | 13 | 11.7 [9.8-17.4] | |
| Yes | 15 | 13.4 [7.8-17.5] | | 10 | 17.0 [15.4-20.2] | | 13 | 13.0 [11.3-26.1] | |
| **Smoking** |  |  | 0.855 |  |  | 0.368 |  |  | 0.761 |
| No | 20 | 11.8 [8.9-16.9] | | 15 | 14.8 [11.1-19.8] | | 20 | 13.9 [10.1-22.8] | |
| Yes | 6 | 14.3 [9.9-15.4] | | 4 | 17.0 [16.5-18.3] | | 6 | 12.0 [10.2-17.9] | |
| **Infection** |  | 0.893 | |  | 0.128 | |  | 0.317 | |
| No | 17 | 13.4 [8.9-16.7] | | 12 | 14.0 [10.6-18.7] | | 18 | 12.2 [9.9-20.3] | |
| Yes | 9 | 12.1 [8.7-14.3] | | 7 | 17.3 [15.8-21.5] | | 8 | 15.2 [11.1-27.5] | |
| **Aneurysm location** |  |  | 0.201 |  |  | **0.009** |  |  | 0.182 |
| Anterior circulation | 18 | 14.3 [9.1-18.3] | | 15 | 17.3 [15.1-21.1] | | 18 | 14.1 [10.7-21.4] | |
| Posterior circulation | 8 | 11.0 [8.4-12.6] | | 4 | 10.0 [8.4-11.5] | | 8 | 10.8 [8.4-17.7] | |
| **Treatment** |  |  | 0.617 |  |  | 0.683 |  |  | 0.269 |
| Clipping | 8 | 11.5 [9.9-20.0] | | 9 | 15.3 [11.2-21.3] | | 9 | 17.4 [11.3-21.7] | |
| Endovascular treatment | 18 | 12.9 [8.6-15.5] | | 10 | 16.2 [13.0-17.7] | | 17 | 11.7 [9.8-20.5] | |
| **Hunt & Hess grade (HH)** |  |  | 0.824 |  |  | 0.933 |  |  | 0.224 |
| Good (HH_1-3_) | 19 | 11.5 [7.7-17.1] | | 12 | 15.4 [12.3-21.0] | | 17 | 12.6 [9.8-26.1] | |
| Poor (HH_4-5_) | 7 | 13.4 [11.5-14.6] | | 7 | 16.8 [12.5-18.3] | | 9 | 13.0 [10.5-20.5] | |
| **Mod. Fisher scale (mFS)** |  |  | 0.402 |  |  | 0.161 |  |  | 0.380 |
| Good (mFS_1-2_) | 7 | 8.9 [6.9-15.5] | | 6 | 13.7 [9.8-15.3] | | 5 | 11.4 [9.8-11.7] | |
| Poor (mFS_3-4_) | 19 | 12.4 [10.4-16.7] | | 13 | 17.3 [13.9-20.9] | | 21 | 14.8 [10.1-21.7] | |
| **Delayed cerebral ischemia (DCI)** |  |  | 0.493 |  |  | **0.026** |  |  | **0.021** |
| no DCI | 10 | 11.0 [8.7-15.4] | | 8 | 11.9 [8.9-15.9] | | 11 | 11.3 [9.2-12.2] | |
| DCI | 16 | 12.9 [9.8-17.8] | | 11 | 17.9 [15.1-22.7] | | 15 | 17.4 [11.7-27.9] | |
| **DCI-related infarction** |  |  | 0.745 |  |  | 0.850 |  |  | 0.624 |
| DCI only | 10 | 13.4 [9.4-16.9] | | 7 | 17.9 [16.3-19.8] | | 10 | 16.2 [11.3-25.1] | |
| DCI-related infarction | 6 | 12.1 [10.4-20.4] | | 4 | 19.6 [14.6-24.9] | | 5 | 26.1 [13.0-29.1] | |
| **Outcome** |  |  | 0.333 |  |  | 0.104 |  |  | 0.614 |
| Favorable (GOSE_5-8_) | 13 | 11.5 [6.9-14.3] | | 10 | 13.7 [9.5-16.5] | | 14 | 11.6 [10.1-19.1] | |
| Unfavorable (GOSE_1-4_) | 9 | 12.1 [10.6-15.7] | | 6 | 17.9 [14.8-22.9] | | 9 | 13.0 [10.5-21.7] | |
| Unknown | 4 | 18.9 [16.0-20.0] | | 3 | 21.3 [18.3-23.2] | | 3 | 19.6 [14.5-25.9] | |

Abbreviations: DCI, delayed cerebral ischemia; GOSE, Glasgow outcome scale – extended; HH, Hunt and Hess grading scale; mFS, modified Fischer scale; 1q, 25^th^ percentile; 3q, 75^th^ percentile. ^a^ total number of patients from which samples were available

**Suppl. Table 2.** Subgroup comparison of MIF levels in cerebrospinal fluid after aSAH.

| **CSF MIF [ng/ml]** | **Early phase (EP_d1-4_)** | | | **Critical phase (CP_d5-15_)** | | | **Late phase (LP_d16-21_)** | | |
| --- | --- | --- | --- | --- | --- | --- | --- | --- | --- |
|  | **n** | **Median**  **[1.q-3.q]** | **p-value** | **n** | **Median**  **[1.q-3.q]** | **p-value** | **n** | **Median**  **[1.q-3.q]** | **p-value** |
| **All patients^a^** | 23 | 31.6 [27.1-33.7] | | 15 | 30.6 [22.1-33.6] | | 11 | 30.0 [29.0-31.8] | |
| **Sex** |  |  | 0.156 |  |  | 0.433 |  |  | 0.838 |
| Male | 8 | 33.7 [29.1-34.2] | | 4 | 33.0 [31.2-33.7] | | 3 | 30.0 [29.8-30.1] | |
| Female | 15 | 30.9 [27.1-31.7] | | 11 | 28.8 [16.6-33.6] | | 8 | 30.8 [27.4-32.1] | |
| **Age** |  |  | **0.049** |  |  | 0.906 |  |  | 0.850 |
| < 59 years | 11 | 27.8 [17.9-32.5] | | 6 | 33.0 [28.2-33.3] | | 7 | 30.0 [29.0-31.0] | |
| ≥ 59 years | 12 | 31.9 [31.5-33.9] | | 9 | 28.8 [18.4-35.0] | | 4 | 30.8 [26.4-32.1] | |
| **Body mass index (BMI)** |  | 0.659 | |  | 0.906 | |  | 0.571 | |
| BMI < 26 | 9 | 31.5 [30.1-34.0] | | 6 | 29.8 [26.1-33.3] | | 7 | 29.8 [29.0-31.0] | |
| BMI ≥ 26 | 14 | 31.6 [22.1-32.3] | | 9 | 30.6 [14.9-35.0] | | 4 | 30.9 [28.5-32.1] | |
| **Arterial hypertension** |  |  | 0.345 |  |  | 0.908 |  |  | 0.715 |
| No | 9 | 33.4 [30.1-34.1] | | 7 | 33.0 [26.3-33.2] | | 6 | 29.9 [29.6-30.2] | |
| Yes | 14 | 31.5 [21.7-32.0] | | 8 | 29.7 [17.5-35.1] | | 5 | 31.7 [24.2-33.0] | |
| **Smoking** |  |  | 0.674 |  |  | 0.470 |  |  | 0.480 |
| No | 17 | 31.5 [26.4-33.4] | | 12 | 31.8 [26.5-33.5] | | 9 | 30.0 [29.5-31.8] | |
| Yes | 6 | 31.8 [28.8-33.6] | | 3 | 14.9 [9.2-25.5] | | 2 | 27.2 [25.7-28.8] | |
| **Infection** |  | 0.423 | |  | 0.695 | |  | 0.102 | |
| No | 16 | 31.7 [27.8-34.0] | | 11 | 28.8 [22.1-33.2] | | 8 | 31.0 [29.9-32.1] | |
| Yes | 7 | 30.9 [24.0-31.9] | | 4 | 32.2 [23.8-34.3] | | 3 | 28.5 [26.3-29.1] | |
| **Aneurysm location** |  |  | 0.789 |  |  | 0.386 |  |  | 0.414 |
| Anterior circulation | 16 | 31.5 [25.9-32.8] | | 12 | 31.8 [24.6-34.1] | | 8 | 29.8 [27.4-31.0] | |
| Posterior circulation | 7 | 31.6 [28.2-34.0] | | 3 | 25.9 [20.3-29.6] | | 3 | 31.7 [30.8-31.8] | |
| **Treatment** |  |  | 0.944 |  |  | 0.346 |  |  | 0.307 |
| Clipping | 6 | 31.2 [28.9-32.1] | | 6 | 32.2 [27.6-34.7] | | 3 | 31.8 [30.1-33.3] | |
| Endovascular treatment | 17 | 31.6 [26.4-34.0] | | 9 | 28.8 [18.4-33.1] | | 8 | 29.9 [28.2-30.6] | |
| **Hunt & Hess grade (HH)** |  |  | 0.423 |  |  | 0.361 |  |  | 0.131 |
| Good (HH_1-3_) | 17 | 31.6 [28.3-34.0] | | 11 | 30.6 [16.6-33.2] | | 8 | 29.9 [28.2-30.7] | |
| Poor (HH_4-5_) | 6 | 29.4 [26.7-31.5] | | 4 | 31.3 [28.3-34.3] | | 3 | 31.7 [30.1-32.4] | |
| **Mod. Fisher scale (mFS)** |  |  | 0.738 |  |  | 0.514 |  |  | - |
| Good (mFS_1-2_) | 7 | 31.6 [30.8-32.9] | | 4 | 28.3 [23.1-31.3] | | 0 |  | |
| Poor (mFS_3-4_) | 16 | 31.2 [24.8-34.0] | | 11 | 33.0 [22.5-34.4] | | 11 | 30.0 [29.0-31.8] | |
| **Delayed cerebral ischemia (DCI)** |  |  | 0.264 |  |  | 1.000 |  |  | - |
| no DCI | 10 | 31.8 [30.5-34.3] | | 5 | 26.7 [25.9-33.3] | | 0 |  | |
| DCI | 13 | 30.9 [26.4-32.4] | | 10 | 31.8 [21.0-33.7] | | 11 | 30.0 [29.0-31.8] | |
| **DCI-related infarction** |  |  | 0.306 |  |  | 0.138 |  |  | 0.414 |
| DCI only | 8 | 28.0 [17.0-32.3] | | 7 | 28.8 [16.5-33.0] | | 8 | 29.9 [28.2-30.7] | |
| DCI-related infarction | 5 | 31.5 [30.9-32.4] | | 3 | 33.9 [32.2-34.8] | | 3 | 31.7 [30.1-33.2] | |
| **Outcome** |  |  | 0.612 |  |  | 0.372 |  |  | 0.796 |
| Favorable (GOSE_5-8_) | 12 | 30.8 [20.0-32.1] | | 7 | 25.9 [16.6-30.9] | | 6 | 30.9 [29.9-31.8] | |
| Unfavorable (GOSE_1-4_) | 7 | 31.6 [29.4-32.2] | | 5 | 30.6 [26.7-33.9] | | 3 | 28.5 [26.3-31.6] | |
| Unknown | 4 | 34.0 [32.6-34.8] | | 3 | 35.0 [34.0-35.5] | | 2 | 29.9 [29.7-30.1] | |

Abbreviations: DCI, delayed cerebral ischemia; GOSE, Glasgow outcome scale – extended; HH, Hunt and Hess grading scale; mFS, modified Fischer scale; 1q, 25^th^ percentile; 3q, 75^th^ percentile. ^a^ total number of patients from which samples were available

**Suppl. Table 3.** Subgroup comparison of MIF levels in microdialysate after aSAH.

| **MD MIF [ng/ml]** | **Early phase (EP_d1-4_)** | | | **Critical phase (CP_d5-15_)** | | | **Late phase (LP_d16-21_)** | | |
| --- | --- | --- | --- | --- | --- | --- | --- | --- | --- |
|  | **n** | **Median**  **[1.q-3.q]** | **p-value** | **n** | **Median**  **[1.q-3.q]** | **p-value** | **n** | **Median**  **[1.q-3.q]** | **p-value** |
| **All patients^a^** | 9 | 7.2 [0.2-15.1] | | 20 | 2.5 [0.3-5.7] | | 8 | 4.2 [1.3-8.1] | |
| **Sex** |  |  | 0.121 |  |  | 0.206 |  |  | 0.275 |
| Male | 1 | <0.1 | | 5 | 0.5 [0.1-3.0] | | 1 | 0.7 | |
| Female | 8 | 9.1 [0.2-17.0] | | 15 | 2.9 [0.6-7.8] | | 7 | 6.4 [1.8-8.7] | |
| **Age** |  |  | **0.071** |  |  | 0.643 |  |  | 0.297 |
| < 59 years | 3 | 0.1 [0.1-3.6] | | 8 | 2.3 [0.4-3.2] | | 3 | 0.7 [0.4-5.3] | |
| ≥ 59 years | 6 | 13.1 [3.0-20.8] | | 12 | 3.1 [0.3-9.7] | | 5 | 6.4 [2.0-7.5] | |
| **Body mass index (BMI)** |  | 0.806 | |  | 0.940 | |  | 1.000 | |
| BMI < 26 | 4 | 3.7 [0.2-11.1] | | 10 | 2.3 [0.2-12.8] | | 4 | 4.5 [1.3-8.1] | |
| BMI ≥ 26 | 5 | 11.0 [0.2-15.1] | | 10 | 2.5 [0.5-4.4] | | 4 | 4.2 [1.5-11.3] | |
| **Arterial hypertension** |  |  | 0.624 |  |  | 0.174 |  |  | 0.655 |
| No | 4 | 11.2 [5.4-17.0] | | 10 | 1.1 [0.2-3.4] | | 5 | 6.4 [2.0-7.5] | |
| Yes | 5 | 0.3 [0.2-11.0] | | 10 | 3.5 [1.2-10.7] | | 3 | 1.5 [0.8-13.7] | |
| **Smoking** |  |  | 0.796 |  |  | 0.275 |  |  | **0.046** |
| No | 6 | 9.1 [1.8-14.1] | | 15 | 3.6 [0.4-10.2] | | 6 | 7.0 [3.1-9.3] | |
| Yes | 3 | 0.3 [0.2-17.4] | | 5 | 0.9 [0.3-2.9] | | 2 | 0.4 [0.2-0.5] | |
| **Infection** |  | 1.000 | |  | 0.190 | |  | 1.000 | |
| No | 5 | 7.2 [0.2-11.0] | | 12 | 0.7 [0.2-3.7] | | 4 | 4.0 [1.3-11.3] | |
| Yes | 4 | 7.7 [0.2-17.0] | | 8 | 3.7 [1.9-10.9] | | 4 | 4.8 [1.5-8.1] | |
| **Aneurysm location** |  |  | 0.242 |  |  | 0.491 |  |  | 0.505 |
| Anterior circulation | 7 | 0.3 [0.1-11.2] | | 17 | 2.9 [0.5-4.5] | | 6 | 1.8 [0.9-7.9] | |
| Posterior circulation | 2 | 16.9 [14.0-19.8] | | 3 | 0.2 [0.1-8.8] | | 2 | 7.0 [6.7-7.3] | |
| **Treatment** |  |  | 0.086 |  |  | 0.290 |  |  | 0.456 |
| Clipping | 5 | 0.2 [0.1-7.2] | | 10 | 2.8 [1.1-9.5] | | 3 | 6.4 [4.2-8.2] | |
| Endovascular treatment | 4 | 16.9 [8.3-25.7] | | 10 | 1.7 [0.2-3.9] | | 5 | 1.5 [0.7-7.5] | |
| **Hunt & Hess grade (HH)** |  |  | 0.245 |  |  | 0.968 |  |  | 1.000 |
| Good (HH_1-3_) | 4 | 16.9 [8.3-25.7] | | 13 | 2.0 [0.3-11.1] | | 5 | 1.5 [0.7-6.4] | |
| Poor (HH_4-5_) | 5 | 0.3 [0.1-7.2] | | 7 | 3.6 [0.9-4.3] | | 3 | 9.9 [6.0-17.9] | |
| **Mod. Fisher scale (mFS)** |  |  | 0.143 |  |  | 0.508 |  |  | - |
| Good (mFS_1-2_) | 1 | 34.6 | | 4 | 1.1 [0.2-5.5] | | 0 |  | |
| Poor (mFS_3-4_) | 8 | 3.7 [0.1-12.1] | | 16 | 3.0 [0.4-5.7] | | 8 | 4.2 [1.3-8.1] | |
| **Delayed cerebral ischemia (DCI)** |  |  | 0.143 |  |  | **0.021** |  |  | - |
| no DCI | 7 | 0.3 [0.1-9.1] | | 6 | 0.2 [0.1-0.7] | | 0 |  | |
| DCI | 2 | 18.9 [17.0-20.8] | | 14 | 3.6 [1.8-10.7] | | 8 | 4.2 [1.3-8.1] | |
| **DCI-related infarction** |  |  | - |  |  | 0.317 |  |  | 0.275 |
| DCI only | 2 | 18.9 [17.0-20.8] | | 9 | 3.0 [0.5-4.5] | | 7 | 2.0 [1.1-7.0] | |
| DCI-related infarction | 0 |  | | 5 | 9.3 [2.0-15.9] | | 1 | 9.9 | |
| **Outcome** |  |  | 0.655 |  |  | 0.101 |  |  | 0.480 |
| Favorable (GOSE_5-8_) | 5 | 0.3 [0.1-22.7] | | 9 | 0.2 [0.2-4.2] | | 4 | 7.0 [5.2-12.1] | |
| Unfavorable (GOSE_1-4_) | 3 | 11.0 [9.1-13.1] | | 7 | 3.6 [2.5-6.9] | | 3 | 2.0 [1.1-6.0] | |
| Unknown | 1 | 0.2 | | 4 | 2.0 [0.8-5.0] | | 1 | 0.7 | |

Abbreviations: DCI, delayed cerebral ischemia; GOSE, Glasgow outcome scale - extended; HH, Hunt and Hess grading scale; mFS, modified Fischer scale; 1q, 25^th^ percentile; 3q, 75^th^ percentile. ^a^ total number of patients from which samples were available
